# Study of a Vocal Feature Selection Method and Vocal Properties for Discriminating Four Constitution Types

**DOI:** 10.1155/2012/831543

**Published:** 2012-03-19

**Authors:** Keun Ho Kim, Boncho Ku, Namsik Kang, Young-Su Kim, Jun-Su Jang, Jong Yeol Kim

**Affiliations:** Division of Constitutional Medicine Research, Korea Institute of Oriental Medicine, 461-24 Jeonmin-dong, Yuseong-gu, Daejeon 305-811, Republic of Korea

## Abstract

The voice has been used to classify the four constitution types, and to recognize a subject's health condition by extracting meaningful physical quantities, in traditional Korean medicine. In this paper, we propose a method of selecting the reliable variables from various voice features, such as frequency derivative features, frequency band ratios, and intensity, from vowels and a sentence. Further, we suggest a process to extract independent variables by eliminating explanatory variables and reducing their correlation and remove outlying data to enable reliable discriminant analysis. Moreover, the suitable division of data for analysis, according to the gender and age of subjects, is discussed. Finally, the vocal features are applied to a discriminant analysis to classify each constitution type. This method of voice classification can be widely used in the u-Healthcare system of personalized medicine and for improving diagnostic accuracy.

## 1. Introduction

Sasang constitution medicine (SCM) provides different treatment methods for the same symptom, as it divides humans into four types (Taeyangin (TY), Soyangin (SY), Tae-eumin (TE), and Soeumin (SE)) according to their appearance and personality [1, 2]. In traditional Korean medicine (TKM), voice inspection forms one of four examinations [3], along with visual inspection [4], auscultation [5], palpation [6, 7], and a survey. Voice inspection has come to the fore as an important theme for studying the four human constitution types [5, 8]. The relation between these constitution types and voice is shown in Sasang-Inhaejinam Saseongron [5] to be as follows: a TY type's voice has a high-pitched tone, derived from good respiratory organs. Their voice is clean and smooth and is matched to the sound of Shang (*商音*) in the traditional Chinese five sounds. A TE type has a loud baritone voice, which therefore sounds heavy, thick, and gentle. Since Yin is superior to Yang, the baritone and the loudness of such a voice are sufficiently high. SY types have poor respiratory organs and thus have a low-pitched tone and light-sounding voice. Their tempo sounds pressing, and their voice can easily spread out widely. Since SE types have a rich voice, they sound lively, slow, and easy. The baritone and loudness of such a voice are sufficiently high like TE types [5].

Based on the TKM relation between the four constitution types and voice, research attempting to scientifically and quantitatively interpret this relation has been conducted. In 2004, Park and Kim [9] acquired significant results from an objective diagnosis using pitch, shimmer, and h1 and h2 harmonics to define differences in voice strength and formant bandwidth for personal identification. In 2005, Kim et al. researched the constitutional characteristics of Korean adult females using pitch, amplitude perturbation quotient (APQ), shimmer, octave, and energy as voice features [10]. In 2006, Kim et al. [11] studied the characteristics of the four constitution types in the voices of 6 to 12 year olds, and Choi et al. researched the significant characteristics of various features of the adult male voice [12].

However, many studies in this field have suggested that it is possible to extract just few voice features and then apply these to statistical parametric estimations. Moreover, they produced meaningless results in constitution classification by adopting various pattern classification methods, as the voice data were obtained from a single site and a limited age interval. In this paper, the extraction of various vocal features of vowels and a sentence in voice data from multiple sites is performed, and the stable vocal features are selected for repeatability in Sections 2.1 and 2.2. The acquisition of independent variables and the removal of outliers for quantitative and reliable discriminant analysis are discussed in Sections 2.3 and 2.5. Moreover, the properties of voice data across all gender and age combinations are obtained, and a general constitution classification method is proposed in Section 2.4.

## 2. Methods and Materials

### 2.1. Voice Acquisition Environment

For the initial voice acquisition, we used personal computers (PCs) and an external sound card to avoid noise from the PC. We selected a sound blaster live 24-bit external soundcard, and used a Sennheiser e-835 s voice recording-only microphone. By using a microphone stand, the distance between the microphone and a subject's mouth was about 5 cm, and the main axis of the microphone cylinder was fixed in order to be parallel to the ground and perpendicular to the mouth. We used GoldWave v5.58 [13] as audio recording software and saved the voice files as a WAV file. To ensure high-quality recordings, a sampling frequency of 44.1 kHz was used.

Each subject was asked to seat themselves comfortably, and speak naturally without tension after taking a sufficient rest more than 1 hour and talking with operators, as though they were the only person in the room, taking care to maintain their usual volume and speed of voice. When pronouncing vowels and sentences, the subject was silent for 1 s at first and then pronounced “a,” “e,” “i,” “o,” and “u” for 3 s, with a 1 s silence between each vowel. Following a further 1 s silence, the subject repeated the given sentence twice, with a 1 s silence between each sentence and at the end of the last sentence to mark the end of the recording. Before this series of experiments commenced, a standard operating procedure (SOP), similar to the acquisition procedure described above, was established to act as a safeguard against differences in individual apparatus operators. All processes then followed this SOP. The constitution types of all subjects were confirmed by SCM doctors in a number of Korean medical clinics after recording the reactions and observing patient improvements following the administration of constitution-specific pharmaceuticals, where the numbers of the clinics and the SCM doctors were 24 and 43, respectively.

### 2.2. Selection of Vocal Features for Pre-Processing

**Figure 1:**
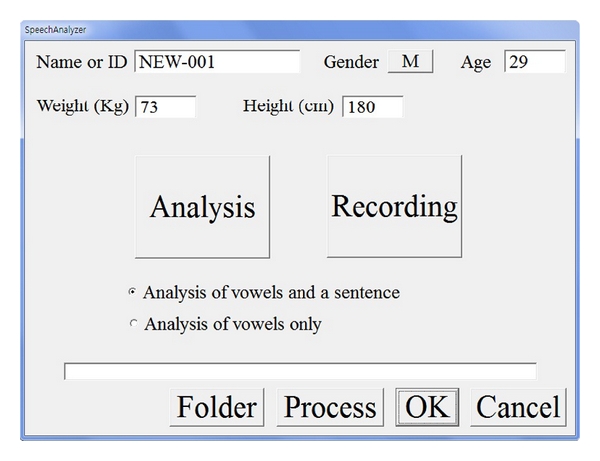
The vocal feature extraction program.

We implemented the C++ program pictured in Figure 1, combined with HTK [14] and Praat [15], to acquire the voice features. The voice features of vowels and a sentence were extracted from the voice wave file captured in the given environment. The window size for the feature extraction was 40 ms, and neighbouring windows were overlapped by 50%. 

**Figure 2:**
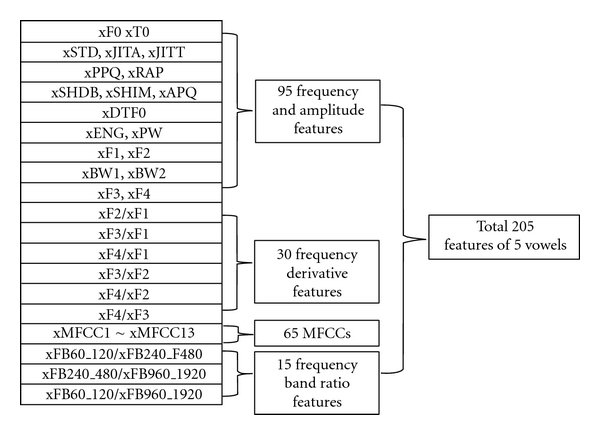
Vocal features of 5 vowels.

**Figure 3:**
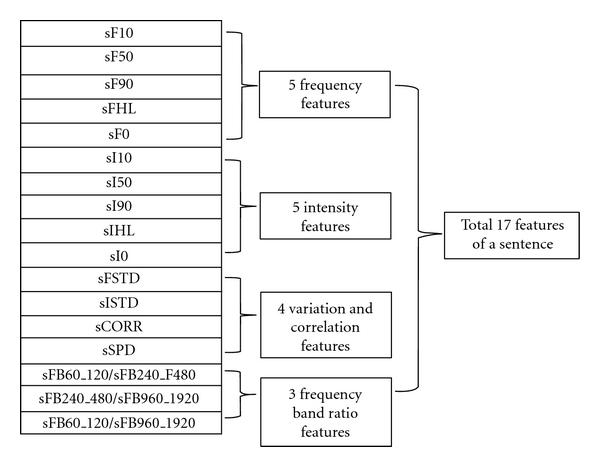
Vocal features of a sentence.

The voice features are shown in Figures 2 and 3 for the five vowels and a sentence, respectively, in order not to lose any vocal information. The features of each vowel are T0, F0 (average pitch frequency), DTF0 (average difference of F0 over the time interval), F1–F4, BW1, BW2 (the formant frequencies and bandwidths of the 1st and 2nd terms) [16], F2/F1, F3/F1, F4/F1, F3/F2, F4/F2, F4/F3 (the ratios of the formant frequencies), JITA, JITT, PPQ, RAP (jitter, percentage of jitter, average variation, and average of pitch frequency), MFCC1-13 (the terms of mel-frequency cepstral coefficient (MFCC)), which are useful in the recognition of voice patterns [17–21], SHDB, SHIM, APQ (shimmer in dB, its variation, and the smooth variation of amplitude), the energy and the power of vowels, and the ratios of voice energies over fixed frequency bands, such as 60–120 Hz, 120–240 Hz, 240–480 Hz, 480–960 Hz, 960–1920 Hz, and 1920–3840 Hz.

The features of a sentence are F10, F50, F90, F0 (the 10th, 50th, and 90th percentiles and the average of pitch frequencies), FHL = (F90 − F50)/(F50 − F10) (the ratio of frequency percentile differences), I10, I50, I90, I0 (the 10th, 50th, and 90th percentiles and the average of intensity), IHL = (I90 − I50)/(I50 − I10) (the ratio of intensity percentile differences), FSTD (variation of the pitch frequency), ISTD (variation of the intensity), SPD (reading speed for a sentence), CORR (Pearson correlation coefficient [22] between F0 and I0 over given intervals), and the ratios of voice energies over the fixed frequency bands, where “s” at the front denotes “sentence.” Finally, we acquired 222 features per subject.

**Figure 4:**
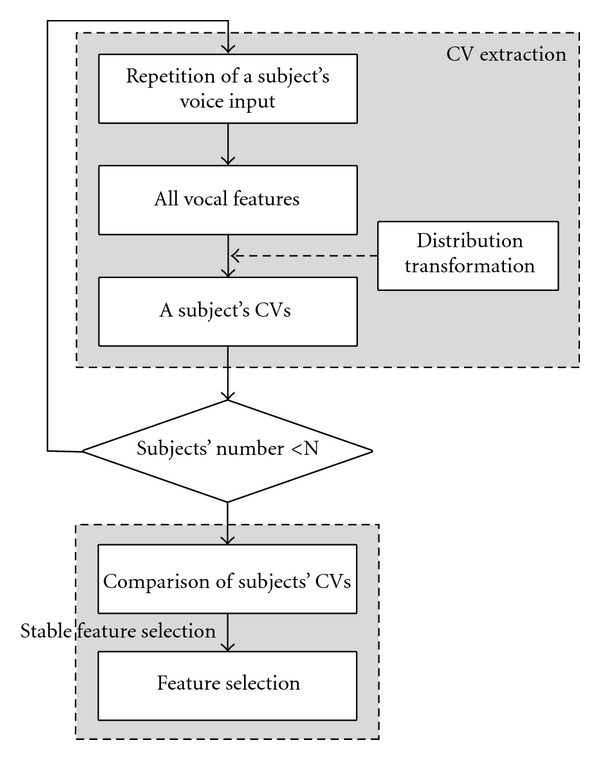
Selection of vocal features.

Among these 222 features, we recognized that some features were sensitive to the variation of a subject's utterance. We attempted to find the stable features by the process shown in Figure 4.

At first, we recorded the vowels and the sentence five times in both the morning and afternoon of the same day, totalling ten recordings from each of six subjects (three males and three female). We then extracted the repeated features of each subject's voice to obtain their CVs (coefficients of variation), and to acquire only the features with sufficient repeatability (defined as those features where the CVs of all six subjects were less than 20%).

**Figure 5:**
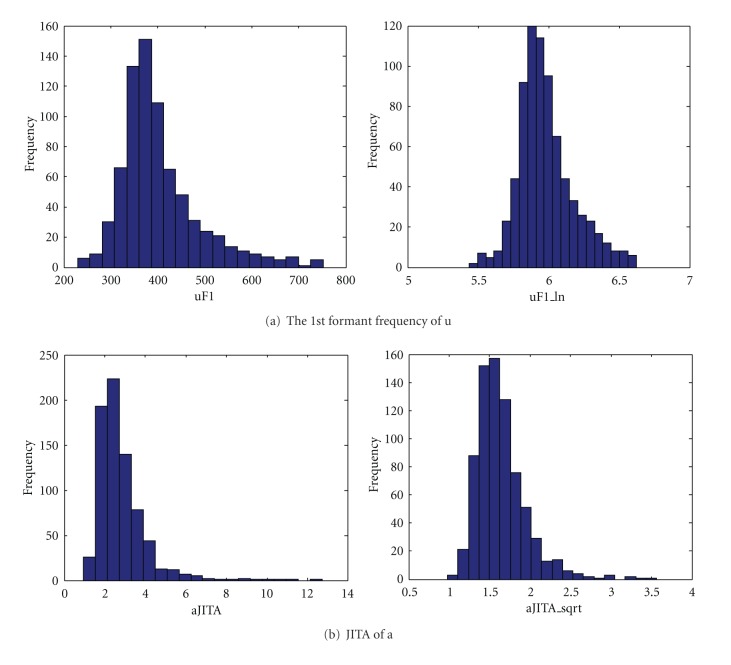
Transformation of variable (feature) distribution.

 However, as shown in the left-hand figures of Figure 5, it was known from precollected datasets that the distributions of a few features were not Gaussian but were instead skewed to the right, which we transformed by taking the log or square root. These transformed distributions are shown on the right of Figures 5(a) and 5(b), respectively, which are similar to a Gaussian model.

Due to the skewed distribution of some features, it was difficult to distinguish between normal data and abnormal data in removing outliers and constructing an exact discriminant function. The variation of the distribution could affect the results from the discriminant function.

### 2.3. Feature Extraction and Correlation Checking between Features

**Figure 6:**
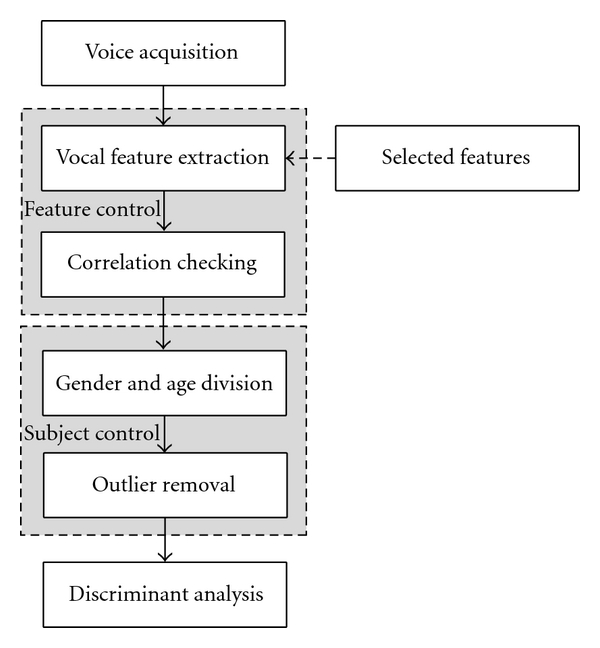
The flow of the overall procedure.

We acquired the voice data and then extracted selected vocal features for preprocessing, according to the flow diagram in Figure 6. Following this, we performed a correlation check, which proceeded as follows. Usually, there are the correlations among some features from the same voice. If two or more predictive variables are highly correlated in the dataset, those variables contain essentially the same information about a response. This phenomenon is called multicollinearity, and it serves to increase standard errors of estimates of the regression coefficients, which can then give confusing and misleading results; the model we construct fits well, even though not every individual feature is statistically significant.

A possible solution to avoid multicollinearity is to eliminate explanatory variables that are redundant to the model by checking the correlation structure of the feature set. One popular method to detect multicollinearity is to calculate the variation inflation factor (VIF) for each of the explanatory variables *x*_*j*_, as

(1) $${\text{VIF}}_{j}=\frac{1}{1-R_{j}^{2}},$$

where *R*_*j*_^2^ is the coefficient of determination of the model including all predictors except the *j*th predictor. If VIF_j_ ≥ 10, then the problem of multicollinearity exists. For our data, some voice features had a very high VIF, and therefore we reduced the data matrix before constructing our classification model.

### 2.4. Division of the Interval on Gender and Age according to the Vocal Properties

**Figure 7:**
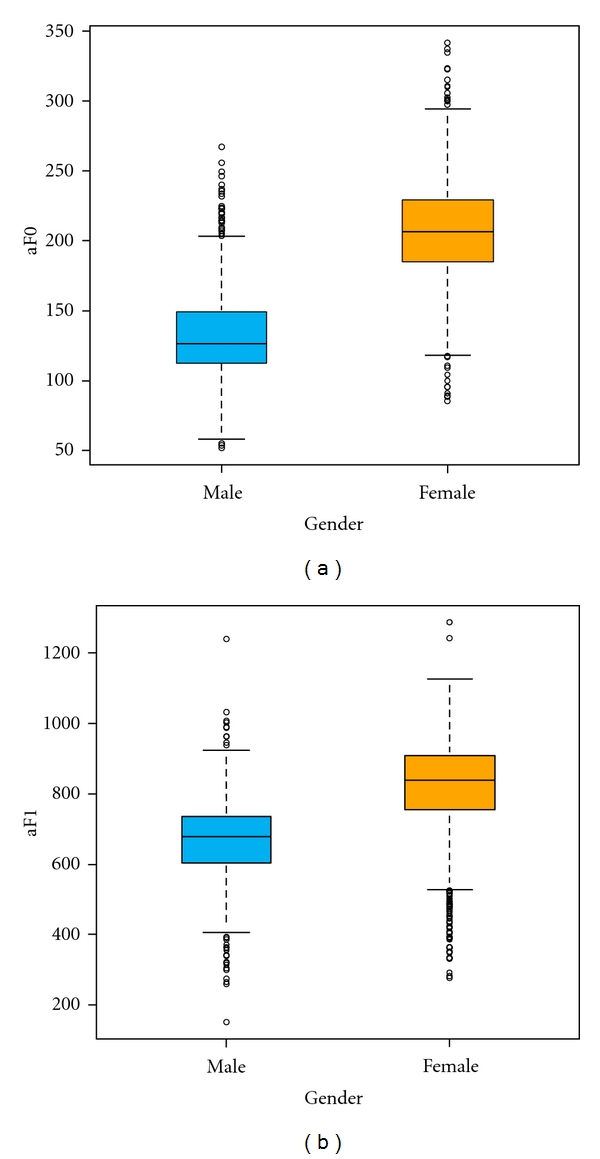
Analysis of the mean difference between voice features (the pitch and the first formant frequency of “a”) according to gender.

**Figure 8:**
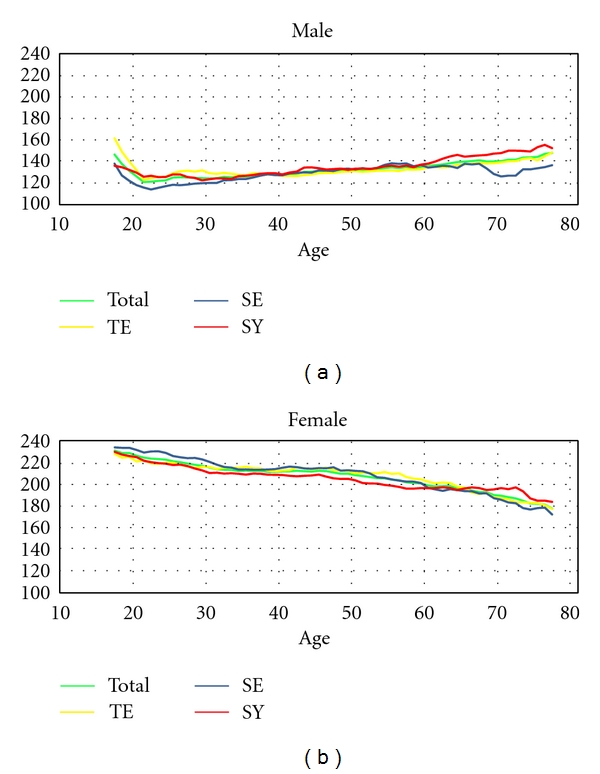
Analysis of the mean of aF0 according to age.

As the next step, the mean and standard deviation of the data were considered. A Student's *t*-test was used to test the significance of gender differences.  Figure 7 shows that the F0 and F1 of females are higher than those of males, and these differences are significant. In addition, Figure 8 illustrates that the F0 of “a” (aF0) in males increases linearly with age from the early twenties to the seventies, and that the aF0s of SY types are larger than those of SE. In females, aF0 decreased linearly from the teenage years until the seventies, and the aF0s of SE types were larger than those of SY before the sixties, but this trend was reversed around the fifties onward. This figure does not show data for the aF0 of TY types because the number of TY types was too small to be analysed statistically (less than 1%). As a result of these factors, we analysed the dataset by excluding the teenagers and dividing the remaining data into age intervals. Although many intervals needed to be discretised further to enable more accurate analysis, we also needed more samples in each interval for a relevant statistical analysis. The interval was divided into only three parts as a trade-off, namely the twenties, the thirties and forties, and the over-fifties, according to gender.

### 2.5. Removal of Outliers

When a feature from an examinee's voice data was outside the range 3 × IQR (interquartile range [23]), the feature was considered to be an outlier and excluded from the analysis, as such data interferes with the accuracy of the discriminant. The remaining data were used to obtain significant features in order to classify the constitution. The outliers were likely to be a result of noise and errors in recording.

### 2.6. Discriminant Analysis

The objective of this study is to determine the vocal features of the frequency-derivative variables, the intensities, and the speed and find the statistical significance of these in relation to the four constitution types. The statistical analysis results will indicate how the four constitution types are associated with the quantitative features. As there are four constitutions (TY, TE, SE, and SY types), it is possible to use a one-way analysis of variance (ANOVA) to test the null hypothesis that there is no difference between the four constitution types. Of the four types, we excluded the classification of TY due to the small number of data available for this type. Statistical analysis was performed with the SPSS Statistical Software (version 14.0) [24], and *P* values of less than 0.05 were considered to be statistically significant.

## 3. Experimental Results

### 3.1. Feature Selection and Data Acquisition

We extracted 92 features per subject (see Table 1), whose CVs in all six subjects were less than 20% and thus had sufficient repeatability as we mentioned in Section 2.2. These features consisted of the pitches, the time period, the log or square root transformation, the ratios of formants and frequency bands for vowels, the percentiles and their ratios of pitch, the intensity, the ratios of frequency bands, and the transformed reading speed for the sentences.

We collected the 2669 voice data from 24 medical centres. Of these, 531 were manually excluded by operators from the analysis, due to being too short, superficially noisy, or having errors in recording time, even if they were acquired in strict accordance with the SOP. The remaining data was composed of 852 males and 1286 females. The numbers of TE, SE, TY, and SY types are 787, 563, 61, and 727, respectively. Data from teenagers with breaking voices were also excluded. The small number of TY types in the dataset would have produced statistically meaningless results, and so they were also excluded from the analysis. The data to be analysed were diversely distributed from the twenties to the seventies, and across both genders. Finally, we analysed 1972 datasets after excluding TYs and teenagers. Initially, the 92 selected features in Table 1 were extracted from these 1972 voice data.

### 3.2. The Correlation between Features and the Representative Features

We calculated the VIF between the selected features, some of which are highly correlated (those with a large VIF value). For the exact discriminant analysis, we should only extract independent representative features from these. The pitches, their derivative variables, and CORR from both the vowels and the sentence are highly correlated with each other. This implies that a similar frequency property is generated from a subject, and thus we only choose uF0_ln and eT0_ln from these. Formants and their ratios from the vowels were highly correlated, and thus we choose only the transformed formants that show a Gaussian distribution. The intensities and their derivatives were reduced to sI0. All the transformed JITAs, that is, the variation of the pitches, remained. The xFB60_120/xFB240_480 and xFB60_120/xFB960_1920 values from all the vowels and the sentence were highly correlated, but the MFCCs did not exhibit significant correlation. As a result, we obtained the representative features given in Table 2, where the number of final features was 38, and their VIFs were each less than 10.

### 3.3. Removal of Outliers

If any one feature from the data of a subject was outside the range 3 × IQR, the data containing this feature were considered to be an outlier and was removed. The number of datasets was reduced to 1923, as detailed in Table 3. The constitutional proportions of the male data are 42.82, 24.07, and 33.11%, and those of the female data are 34.93, 28.18, and 36.89%, for TE, SE, and SY, respectively.

### 3.4. Classification of Constitution

Due to the different vocal characteristics according to gender and age, we were able to divide the subjects into six groups, which were a combination of the two gender and three age intervals. 

First, we statistically extracted the significant features of each group. Secondly, a discriminant function was generated from these significant features. Finally, the classification of constitution types with their different discriminant functions was performed. This procedure was repeated for the six groups. 

Although there are four constitution types, the number of TY types was too small to be statistically analysed. Therefore, after excluding the TY data from our analysis, we classified the three remaining types. The results of the constitution accuracies are shown in Table 4. For males in their twenties, the constitution accuracies were 52, 53, and 44% for TE, SE, and SY, respectively, and the overall average was 50%. For males in their thirties and forties, accuracies of 42, 58, and 52% for TE, SE, and SY were found, respectively, and the average was again 50%. For males of more than fifty years of age, the constitution accuracies were 54, 55, and 46% for TE, SE, and SY, respectively, with an overall average of 51%. 

For females in their twenties, the constitution accuracies were 55, 38, and 49% for TE, SE, and SY, respectively, and the overall average was 53%. For females in their thirties and forties, they were 42, 50, and 45% for TE, SE, and SY, respectively, with an overall average of 46%. For females of more than fifty years of age, they were 40, 50, and 54% for TE, SE, and SY, respectively, with an overall average of 47%. 

The same tendency towards higher accuracy rates in SE types than in TE and SY types was observed in males and females in all age intervals. Females in their twenties displayed a higher accuracy rate than the other female age groups, and the average accuracy rates of males and females were similar to each other. 

## 4. Discussion and Conclusions

This study aimed to utilize scientific and systematic methods to detect significant voice features to help determine the constitution type of a patient. Therefore, we attempted a classification procedure in order to objectively and quantitatively distinguish the constitution type by analysing the characteristics of subjects' voices free from noise and error. 

To find stable and significant vocal features, we employed CVs and VIFs as feature selection methods. CV threshold of 20% was used for selecting stable features, and then VIF threshold of 7-8 was used for reducing the correlation between features. Only one correlated feature was selected and analysed for the classification procedure. We found a close correlation between the pitches of vowels and a sentence, the formant frequencies and their ratios of vowels, and the ratios of frequency band for each vowel or each sentence, which show the frequency properties. In addition, the average and median intensities of the sentence were closely correlated. By avoiding the explanatory variables, we obtained accurate discriminant functions from 38 feature variables. The discriminant function was made using vocal features, including transformed ones, to reduce the correlation according to the gender and age interval as follows. Generally, among the vowels, a is a low tone, e is an intermediate tone, and i, o, and u are relatively high tones. For males in their twenties, TE gave a larger value than SY on the F2 of the low-pitched a and had a low-pitch property. SE had larger weight on the F3 and F4 of an intermediate pitched e and did not seem to belong to high or low tones. 

For females in their twenties, SY types were larger than TE on the F4 of the high-pitched i, as well as the F3 of the high-pitched o. In particular, TE on the iFB240_480/iFB960_1920 showed a low tone as a low value. In addition, since SY showed a smaller pitch variation for the letter o, this implies that SY types pronounced this letter more clearly. 

For males in their thirties and forties, SE was larger on the F2 of the intermediate pitched e, and SY was larger on the F4 of the high-pitched u. TE showed a low-pitched property on the frequency band ratio (oFB240_480/oFB960_1920) of o, and the pitch variations of the high-pitched u for SY and SE were smaller, which showed that their voices are clearer. 

For females in their thirties and forties, the strength (sI0) of SE on the sentence was significant, which is an important factor distinguishing SE types. In addition, TE was larger on the pitch variation of the high-pitched o, which showed that TE types have rough voices. 

For males above the age of fifty, SY was larger than other types on the F1 of the high-pitched o. The low-pitched property of TE types and the high-pitched one of SY types were shown on the frequency band of o and u, respectively. 

For females above the age of fifty, SY was larger on the F1 of the high-pitched a, and SE and SY types showed some relation with the strength (sI0) of their utterance. It was known that factors distinguishing TE were not large in this age interval. 

From the preceding comments, we can deduce that the variables contributing to the determination of each constitution type differ according to gender and age. 

The MFCCs are often used in voice recognition, but these were underutilized in the discriminant functions for distinguishing the four constitution types. However, the transformed formant frequencies were utilized in every age interval and gender, and these parameters reflect the structure of the vocal tract through the oral and nasal cavities for articulation—the velum, jaw, tongue, and lips. 

In this study, we extracted the stable vocal features of the voice data, transformed these features, and divided the data according to the vocal properties of gender and age. We then minimized the correlated features, removed outliers, and developed discriminant functions that are adaptive to gender and age from the features to show the reported accuracy. 

Finally, the discriminant functions gave an accuracy of about 50% in classifying the constitution for every age interval and every gender. This accuracy level is meaningful, as it was determined using only the voice. If there are three types of constitution, the probability of one type being chosen at random is only 1/3. Moreover, if integrative algorithms containing face and body shape data and a survey, as well as voice data, are developed for the classification of constitution, then our results for voice classification can contribute to the improvement of this accuracy level. 

In future, we need to find more effective vocal features for classification and suggest the discriminant method to distinguish health conditions as well as the constitution type. In particular, the voice analysis method will play a critical and essential role, eventually leading to a system for u-Healthcare and smart phones.

## Figures and Tables

**Table 1:** 92 features selected as having less than 20% CV in each of the six subjects.

Features			
aF0	oJITT	sF10	uF1_ln
aF2/aF1	oF2/oF1	sF50	aF2_ln
aF3/aF1	oF3/oF1	sF90	eF2_ln
aF4/aF1	oF4/oF1	sFHL	iF2_ln
aF3/aF2	oF3/oF2	sI10	oF2_ln
aF4/aF2	oF4/oF2	sI50	uF2_ln
aF4/aF3	oF4/oF3	sI90	aF3_ln
eF0	uT0	sIHL	eF3_ln
eF2/eF1	uPPQ	sF0	iF3_ln
eF3/eF1	uF2/uF1	sFSTD	oF3_ln
eF4/eF1	uF3/uF1	sI0	uF3_ln
eF3/eF2	uF4/uF1	sISTD	aF4_ln
eF4/eF2	uF3/uF2	iFB240_480/iFB960_1920	eF4_ln
eF4/eF3	uF4/uF2	oFB240_480/oFB960_1920	iF4_ln
iF0	uF4/uF3	uFB240_480/uFB960_1920	oF4_ln
iT0	aMFCC13	sFB60_120/sFB240_480	uF4_ln
iF2/iF1	eMFCC2	sFB240_480/sFB960_1920	eT0_ln
iF3/iF1	eMFCC13	sFB60_120/sFB960_1920	sSPD_ln
iF4/iF1	iMFCC2	uF0_ln	aJITA_sqrt
iF3/iF2	iMFCC13	aF1_ln	eJITA_sqrt
iF4/iF3	oMFCC6	eF1_ln	iJITA_sqrt
oF0	oMFCC13	iF1_ln	oJITA_sqrt
oT0	uMFCC13	oF1_ln	uJITA_sqrt

**Table 2:** Analytic results from the VIF calculation of vocal features.

Variable	VIF	Variable	VIF
uF0_ln	4.94	oF4_ln	2.39
aF1_ln	3.12	uF4_ln	2.13
eF1_ln	2.96	eT0_ln	5.68
iF1_ln	1.87	eMFCC13	3.77
oF1_ln	3.30	eMFCC2	6.38
uF1_ln	2.40	iMFCC2	6.26
aF2_ln	2.52	oMFCC6	1.88
eF2_ln	2.86	sI0	3.52
iF2_ln	2.05	sSPD_ln	1.08
oF2_ln	1.96	aFB60_120/aFB240_480	2.52
uF2_ln	2.44	iFB240_480/iFB960_1920	2.42
aF3_ln	1.95	oFB240_480/oFB960_1920	4.12
eF3_ln	4.04	uFB240_480/uFB960_1920	3.38
iF3_ln	2.29	sFB240_480/sFB960_1920	2.33
oF3_ln	2.05	aJITA_sqrt	2.81
uF3_ln	2.07	eJITA_sqrt	2.95
aF4_ln	2.55	iJITA_sqrt	1.76
eF4_ln	3.41	oJITA_sqrt	1.34
iF4_ln	2.71	uJITA_sqrt	1.23

**Table 3:** Distribution of datasets after removal of outliers.

	TE	SE	SY	Total
Male	322 (42.82%)	181 (24.07%)	249 (33.11%)	752

Female	409 (34.93%)	330 (28.18%)	432 (36.89%)	1171

	731	511	681	1923

**(a):** The coefficients of the linear discriminant function for subjects in their 20 s.

Male	SC			Female	SC		
	TE	SE	SY		TE	SE	SY

uF1_ln	43.40	44.45	42.78	aF1_ln	42.20	43.02	41.15
aF2_ln	811.02	813.80	806.72	aF2_ln	233.56	236.08	234.27
iF2_ln	454.78	446.43	451.97	iF2_ln	−119.84	−121.15	−120.32
eF3_ln	1263.15	1272.38	1270.05	oF3_ln	601.51	600.09	604.50
eF4_ln	2682.67	2691.38	2690.17	uF3_ln	921.89	928.00	925.54
* *	* *	* *		iF4_ln	1700.96	1707.70	1703.23
* *	* *	* *		uF4_ln	2233.97	2226.67	2230.50
* *	* *	* *		eT0_ln	300.49	297.49	301.75
* *	* *	* *		oMFCC6	−3.19	−3.16	−3.15
aFB60_120/aFB240_480	−40.32	−39.39	−40.48	iFB240_480/iFB960_1920	163.16	161.13	161.99
oFB240_480/oFB960_1920	22.25	21.12	21.06	sFB240_480/sFB960_1920	−76.00	−74.78	−75.67
* *	* *	* *		oJITA_sqrt	4.24	3.32	2.74
(Constant)	−20662.87	−20765.44	−20718.69	(Constant)	−23380.96	−23416.62	−23413.53

**(b):** The discriminant results for subjects in their 20 s.

* *	Male					Female				
FC	Prediction			Total	Accuracy	Prediction			Total	Accuracy
	TE	SE	SY			TE	SE	SY		

TE	61	25	31	117	0.52	65	31	23	119	0.55
SE	14	38	20	72	0.53	19	57	23	99	0.58
SY	25	21	36	82	0.44	32	35	64	131	0.49

Average	* *	* *	* *	* *	0.5	* *	* *	* *	* *	0.53

**(c):** The coefficients of the linear discriminant function for subjects in their 30 s/40 s.

Male	SC			Female	SC		
	TE	SE	SY		TE	SE	SY

uF1_ln	85.30	85.85	83.25	aF1_ln	1.43	2.31	3.13
eF2_ln	894.33	901.15	892.94	uF1_ln	202.00	200.27	201.95
uF3_ln	477.98	476.34	474.32	aF2_ln	531.59	533.79	531.03
eF4_ln	1528.36	1523.34	1529.25	uF4_ln	4378.71	4383.83	4380.97
uF4_ln	208.76	209.21	213.63	* *	* *	* *	* *
* *	* *	* *	* *	sI0	4.11	4.15	4.10
oFB240_480/oFB960_1920	11.09	10.69	10.39	sFB240_480/sFB960_1920	69.47	69.80	70.48
eJITA_sqrt	−79.09	−77.53	−77.82	oJITA_sqrt	−19.49	−20.20	−19.70
uJITA_sqrt	−9.09	−10.98	−10.24	* *	* *	* *	* *
(Constant)	−12602.18	−12604.26	−12595.92	(Constant)	−20883.77	−20939.87	−20912.08

**(d):** The discriminant results for subjects in their 30 s/40 s.

* *	Male					Female				
FC	Prediction			Total	Accuracy	Prediction			Total	Accuracy
	TE	SE	SY			TE	SE	SY		

TE	40	27	28	95	0.42	63	40	46	149	0.42
SE	14	38	13	65	0.58	32	71	38	141	0.50
SY	19	22	45	86	0.52	46	56	83	185	0.45

Average	* *	* *	* *	* *	0.50	* *	* *	* *	* *	0.46

**(e):** The coefficients of the linear discriminant function in subjects over 50 years old.

Male	SC			Female	SC		
	TE	SE	SY		TE	SE	SY

oF1_ln	206.57	203.55	206.83	eF1_ln	214.50	214.33	216.28
aF2_ln	726.79	729.70	729.64	iF1_ln	313.68	313.04	315.48
oF3_ln	1157.23	1155.75	1153.21	iF2_ln	167.21	169.08	166.61
* *	* *	* *	* *	eF3_ln	699.49	698.85	701.71
* *	* *	* *	* *	uF3_ln	1452.33	1447.92	1450.98
oMFCC6	−0.69	−0.74	−0.68	* *	* *	* *	* *
* *	* *	* *	* *	sI0	0.46	0.49	0.49
oFB240_480/oFB960_1920	25.36	24.27	24.66	aFB60_120/aFB240_480	100.48	100.49	101.74
uFB240_480/uFB960_1920	284.86	285.94	283.19	* *	* *	* *	* *
sFB240_480/sFB960_1920	53.43	55.70	55.19	* *	* *	* *	* *
(Constant)	−8441.34	−8440.88	−8428.15	(Constant)	−10873.65	−10844.66	−10900.15

**(f):** The discriminant results in subjects over 50 years old.

	Male					Female				
FC	Prediction			Total	Accuracy	Prediction			Total	Accuracy
	TE	SE	SY			TE	SE	SY		

TE	59	29	22	110	0.54	56	43	42	141	0.4
SE	11	24	9	44	0.55	23	45	22	90	0.5
SY	22	22	37	81	0.46	25	28	63	116	0.54

Average					0.51					0.47
